# RNAi Efficiency, Systemic Properties, and Novel Delivery Methods for Pest Insect Control: What We Know So Far

**DOI:** 10.3389/fphys.2016.00553

**Published:** 2016-11-17

**Authors:** Mallikarjuna R. Joga, Moises J. Zotti, Guy Smagghe, Olivier Christiaens

**Affiliations:** ^1^Department of Crop Protection, Faculty of Bioscience Engineering, Ghent UniversityGent, Belgium; ^2^Department of Crop Protection, Molecular Entomology, Federal University of PelotasPelotas, Brazil

**Keywords:** RNA interference (RNAi), systemic RNAi, delivery, uptake, pest control

## Abstract

In recent years, the research on the potential of using RNA interference (RNAi) to suppress crop pests has made an outstanding growth. However, given the variability of RNAi efficiency that is observed in many insects, the development of novel approaches toward insect pest management using RNAi requires first to unravel factors behind the efficiency of dsRNA-mediated gene silencing. In this review, we explore essential implications and possibilities to increase RNAi efficiency by delivery of dsRNA through non-transformative methods. We discuss factors influencing the RNAi mechanism in insects and systemic properties of dsRNA. Finally, novel strategies to deliver dsRNA are discussed, including delivery by symbionts, plant viruses, trunk injections, root soaking, and transplastomic plants.

## Introduction

Over two and a half decades ago, the silencing ability of antisense RNA was first described in the nematode *Caenorhabditis elegans* (Fire et al., 1991). The interfering mediator was afterwards determined as being a double-stranded RNA (dsRNA), rather than a single-stranded antisense RNA (Fire et al., 1998). The phenomenon of RNA interference (RNAi) as a method for gene silencing has allowed unique advancements in the understanding of gene function in many organisms and thus accelerated the use of reverse genetics to new levels. The application of this technology did not go unnoticed in agriculture, where since then crop protectors have been seeking for its practical application in insect management (Gordon and Waterhouse, 2007; Price and Gatehouse, 2008; Zotti and Smagghe, 2015). During the following years, further advancements on several fronts such as design, synthesis and delivery of dsRNA led to the development of RNAi-based applications for plant protection and therapeutics (Gordon and Waterhouse, 2007; Huvenne and Smagghe, 2010; Palli, 2014; Zotti and Smagghe, 2015).

Systemic RNAi refers to the principle that dsRNA uptake via injections, soaking or feeding initiates a whole body and persistent suppression of mRNA from the target gene. This principle entails uptake of dsRNA from the environment and subsequent transport of the RNAi signal between cells and tissues in the body. To date, a reasonable understanding toward this process in insects remains elusive and still precludes several potential practical applications for insect pest control. From a pest control perspective, the absence of a functional systemic RNAi system results in ineffective knockdown or a knockdown with merely a localized effect (i.e., midgut where dsRNA uptake occurs), which may or may not cause mortality. Although RNAi acts following a general conserved strategy, some components can radically change depending on the taxonomic kingdom or group, especially regarding to the molecular mechanism behind cellular uptake and systemic spread of silencing (Terenius et al., 2011; Burand and Hunter, 2013; Gu and Knipple, 2013; Scott et al., 2013; Zotti and Smagghe, 2015).

While strict and laborious regulatory rules from protection agencies may hamper GM crop releases, non-transformative RNAi strategies with similar results, such as bacterial production of dsRNA, dsRNA uptake by plant roots through soil irrigation in rooted seedlings and trees, trunk delivery by plant cuttings or injection into woody plants have shown encouraging results (Hunter et al., 2012; Scott et al., 2013).

In the present work, we first discuss the current knowledge of minimum requirements for efficient RNAi in insects followed by an overview of the systemic proprieties of dsRNA. Second, we discuss what can be done to improve RNAi efficiency in relatively recalcitrant species. Finally, novel delivery methods including non-transformative are discussed in light of the current knowledge and technology.

## RNAi pathways and its components: a general overview

**Figure 1:**
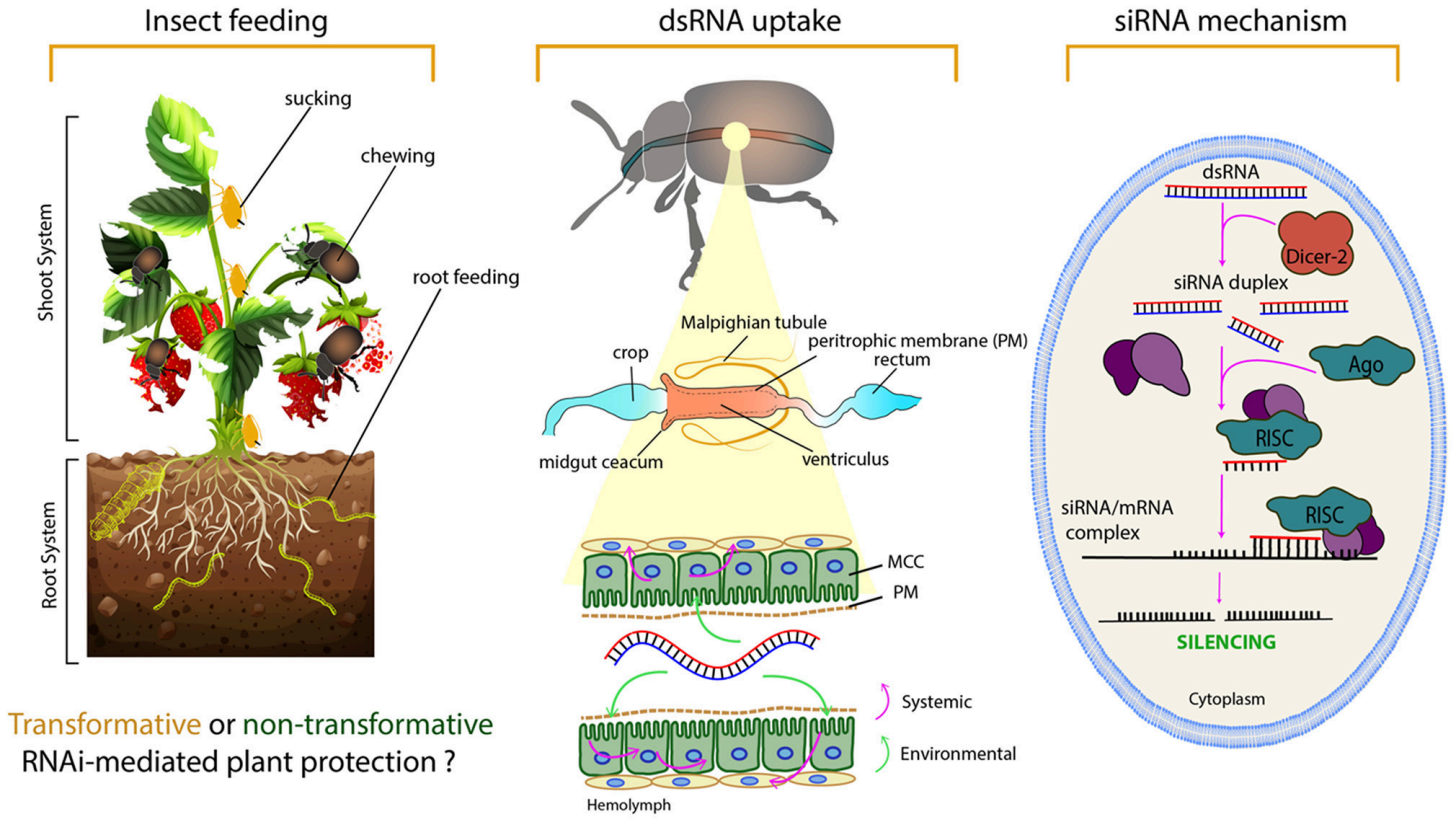
**The basic levels of RNAi from an insect control perspective**. **The left panel** demonstrates some questions that need to be taken into consideration regarding insect feeding behavior, using a hypothetical example of a strawberry plant and some pest insects. Knowing the feeding habits of the target insect is important in planning the (delivery) strategy and whether a transformative or non-transformative RNAi-plant protection approach might be preferred. For chewing insect, dsRNA can be taken up directly from leaves after dsRNA has been delivered through a foliar spray such as a normal plant protectant chemical. Sprayable RNAi-based biocontrol products are in the process of development and will soon be on the market. However, spraying method would not influence the piercing-sucking pests which fed on phloem sap, insects that fed on root system or stem borer pests which fed in the plant stems. For sap-sucking insects, the dsRNA needs to be delivered through the phloem sap, which can be achieved via irrigation water, trunk injection for perennial trees, *in planta* dsRNA production (transgenic or transplastomic plants) or recombinant plant viruses (further details on dsRNA delivery approaches are provided latter). **The middle panel** illustrates the dsRNA path/uptake by the microvilli of the columnar cells (MCC) in the insect midgut, as well as its environmental and systemic properties. **The right panel** shows the cellular siRNA mechanism of gene silencing.

Three major RNAi pathways have been characterized so far: the microRNA (miRNA), piwiRNA (piRNA), and small interfering RNA (siRNA) pathways. The application of RNAi technology for pest control is based on the introduction of dsRNA into the insect body to silence a gene of interest, thereby activating the siRNA pathway. In brief, upon entry into the cell, the exogenous dsRNA is processed by a ribonuclease III enzyme, called Dicer-2, into small interfering RNAs (siRNAs). These 21–24 nucleotide duplexes are subsequently incorporated in the so-called RNA-induced silencing complex (RISC) where the duplex is unwound. Subsequently, an Argonaute2 (AGO2) protein cleaves the passenger (sense) strand and the guide (antisense) strand remains connected with the RISC. Afterwards, the guide strand of the siRNA guides the RISC and allows Watson-Crick base pairing of the complex to complementary target mRNA for cleavage of target mRNA by AGO2 protein. By this degradation of the target mRNA, specific post-transcriptional gene silencing occurs (Agrawal et al., 2003; Pecot et al., 2011; Figure 1 Right panel).

## What matters for RNAi efficiency?

RNAi technology has demonstrated its potential to control insect pests. However, the efficiency of RNAi can vary greatly between the different insect orders. In many RNAi recalcitrant insect species, the gene knockdown is around 60% or lower and silencing is often temporary (Huvenne and Smagghe, 2010; Li et al., 2013). In contrast, the gene knockdown in RNAi sensitive coleopterans is often 90% or higher, requires only very small doses and the effect can be long lasting and even hereditary (Baum et al., 2007; Zhu et al., 2011; Bolognesi et al., 2012; Rangasamy and Siegfried, 2012). This evidence clearly suggests that some barriers are influencing RNAi efficiency in insects.

### Systemic properties and dsRNA uptake

Systemic RNAi is determined by the systemic spread of a silencing RNAi signal inside the body of an organism. There are two types of RNAi: cell-autonomous RNAi and non-cell-autonomous RNAi (Whangbo and Hunter, 2008; Huvenne and Smagghe, 2010). Cell-autonomous RNAi refers to RNAi that happens inside the cell while non-cell-autonomous RNAi entails uptake into the cell and/or transport of the silencing signal from one cell to another and from one tissue to another. Non-cell-autonomous involves the phenomenon of environmental RNAi, which triggers the RNAi by environmental exposure either by soaking or feeding (Baum and Roberts, 2014). Although RNAi pathways share mostly the same elements among insect species, its systemic nature, if present, may act by different molecular mechanisms across different insect taxa. This is exemplified by the differences that are observed between different insect orders regarding the Sid-mediated uptake and the presence of *Sid*-genes in the genomes.

Two different pathways have been described for dsRNA uptake in insect pests. These are the transmembrane Sid-1 channel protein-mediated pathway and the endocytic pathway. In nematodes, the *Sid-2* gene encodes a membrane protein, which is situated in the intestinal cells. The protein Sid-2 imports dsRNA from the intestinal lumen (Winston et al., 2007; McEwan et al., 2012) through endocytosis and exports the silencing RNAs to other neighboring cells through Sid-1 channels from the internalized vesicles by way of passive movement (Whangbo and Hunter, 2008; McEwan et al., 2012). Therefore, environmental RNAi needs cooperation between Sid-1 and Sid-2 proteins. The *Sid-1* genes seem to be present in most insect species, but so far no *Sid-2* genes have been found in insect species whose genomes have been sequenced (e.g., Tomoyasu et al., 2008; Xu and Han, 2008; Zha et al., 2011; Cappelle et al., 2016). A phylogenetic analysis suggested that *Sid-1* like genes in *Tribolium* may not be orthologous to *Sid-1*, but rather to the *C. elegans Tag-130* gene which is not associated in systemic RNAi in nematodes (Tomoyasu et al., 2008). We will therefore name them Sid-1-like channel proteins henceforth. These Sid-1-like channel proteins have been shown to be involved in dsRNA uptake in some insect species, such as the brown planthopper [BPH, *Nilaparvata lugens* (Xu et al., 2013)], the Colorado potato beetle [CPB, *Leptinotarsa decemlineata* (Cappelle et al., 2016)] and the red flour beetle *Tribolium castaneum* (Tomoyasu et al., 2008). The number of *Sid-1*-like genes has been found to vary between insects belonging to different orders (Table 1). Insects in most orders seem to have only one *Sid-1*-like gene. However, in the genome of several coleopteran insects, 2 or even 3 *Sid-1*-like genes have been identified (Tomoyasu et al., 2008; Miyata et al., 2014). In contrast, dipterans such as *D. melanogaster* seem to lack *Sid-1*-like genes altogether in their genome.

**Table 1:** **Overview of reported dsRNA uptake in nematode and insects**.

**Organism**	**Order**	**No. of Sid-1 homologous**	**Sid-1 involved**	**Endocytosis involved**	**Oral RNAi sensitive**	**Injection RNAi sensitive**	**Environ-mental RNAi**	**Systemic RNAi**	**Environmental RNAi**
*Caenorhabditis elegans*	Rhabditida	1		N.D	H.S	H.S	+ +	+ +	Winston et al., 2002; Nuez and Félix, 2012; Sim and Hibberd, 2016
*Tribolium castaneum*	Coleoptera	3			V	H.S	+	+ +	Tomoyasu et al., 2008; Xiao et al., 2015; El Halim et al., 2016
*Leptinotarsa decemlineata*		2			H.S	H.S	+ +	+ +	Zhu et al., 2011; Cappelle et al., 2016; Shukla et al., 2016
*Diabrotica v. virgifera*		2		N.D	H.S	H.S	+ +	+ +	Alves et al., 2010; Miyata et al., 2014; Li et al., 2015b
*Bombyx mori*	Lepidoptera	3		N.D	IS	V	−	+	Tomoyasu et al., 2008; Liu et al., 2013
*Schistocerca gregaria*	Orthoptera	1			IS	H.S	−	+ +	Van Wielendaele et al., 2013; Wynant et al., 2014a
*Locusta migratoria*		1		N.D	IS	H.S	−	+ +	Luo et al., 2012, 2013
*Apis mellifera*	Hymenoptera	1		N.D	S	S	+	+	Aronstein et al., 2006; Jarosch and Moritz, 2011; Garbian et al., 2012
*Drosophila melanogaster*	Diptera	0			V/IS	S	+	+	Saleh et al., 2006; Ulvila et al., 2006; Berry et al., 2009; Garlapow et al., 2015
*Bactrocera dorsalis*		0			V	S	+	+	Chen et al., 2008; Li et al., 2015c; Zheng et al., 2015
*Nilaparvata lugens*	Hemiptera	1		N.D	IS	S	−	+	Xu et al., 2013; Zhang et al., 2013; Zhao et al., 2016

*N.D, Not Determined; Green color, Yes; Red color, No; H.S, Highly Sensitive; S, Sensitive; V, Variable; IS, Insensitive; +, Present but not robust; ++, Present and robust; −, Not present (simple modifications from Cappelle et al., 2016)*.

Studies in *Drosophila* S2 cells have confirmed the speculation of dsRNA uptake through the endocytic pathway. Pattern recognition receptors (PRRs) are important for the uptake of dsRNA by receptor-mediated endocytosis. These receptors play a key role in the phagocytosis of several pathogens, but are structurally different (Ulvila et al., 2006). When these two scavenger receptors had been silenced simultaneously with RNAi in *D. melanogaster*, the uptake of dsRNA diminished with more than 90% (Ulvila et al., 2006). This suggested that in *D. melanogaster*, the uptake of dsRNA relies on receptor-mediated endocytosis. Most of the studies examining dsRNA uptake so far focused on either the Sid-1-like dependent system or the endocytic pathway, preventing a comprehensive assessment of the involvement of these pathways on dsRNA uptake of various insect species. However, Cappelle et al. (2016) have recently proven that in the coleopteran CPB both the Sid-1-like channel proteins as well as the receptor-mediated endocytosis are involved in dsRNA uptake. In contrast to uptake of dsRNA, no information is available yet on the transport of dsRNA within the insect body and which system is involved in this. What we do know however is that the systemic RNAi system as it exists in nematodes, is not present in insects since *Sid-1* and *Sid-2* homologs are not found in insect genomes. Another major difference between insects and nematodes is found when we look at the amplification of the RNAi system. In *C. elegans*, secondary siRNAs are created via an RNA-dependent RNA polymerase (RdRP) system, which amplifies and prolongs the silencing effect. In insects, no clear homologs for this RdRP has been discovered yet. However, this does not necessarily mean that insects do not have a similar amplification system, as it can be based on a different enzyme with a similar working mechanism as RdRP, or a completely distinct mechanism that still remains to be unraveled. Indeed, in some species, for example some coleopterans, the RNAi effect is so strong and can last so long that it would be likely that such a system is present in these insects. On the other hand, many other insects do require large amounts of dsRNA to elicit a moderate effect, which is often short lived.

Uptake of dsRNA by the epithelial cells of the insect midgut is critical to the effectiveness of RNAi response. The brush border membrane topography of the insect midgut allows the uptake of supplements (vitamins and minerals). It is unclear to what degree the perimicrovillar membrane in the midgut of Hemiptera species (Silva et al., 2004) or the peritrophic matrix in the midgut of Coleoptera and Lepidoptera species (Lehane, 1997; Hegedus et al., 2009) acts as a physical barrier to the delivery of dsRNA. After arrival of dsRNA to the gut membrane surface, the epithelial cells must take up the dsRNAs from the surface of gut membrane and convey these to the intracellular RNAi machinery. Whereas, the route of cellular uptake associates with endocytosis, the discharge or escape from the endosome turns into a critical step to transfer the dsRNA to the cytoplasm (Varkouhi et al., 2011).

### Nucleases and viruses

The rapid clearance and degradation of circulating dsRNA (Thompson et al., 2012; Christensen et al., 2013) limit the potential for ingested dsRNA to trigger the RNAi mechanism. In general, dsRNA is stable, much more so than single-stranded RNA, but it must be rapidly taken up in the cells and digested into siRNA by Dicer-2 (Katoch and Thakur, 2012). The presence of salivary nucleases in the midgut can quickly degrade the ingested dsRNA molecules, which is considered to be an important barrier for RNAi efficiency (Furusawa et al., 1993; Arimatsu et al., 2007a,b; Rodríguez-Cabrera et al., 2010; Terenius et al., 2011; Liu et al., 2013; Luo et al., 2013; Christiaens et al., 2014; Wynant et al., 2014b). The existence of dsRNases in the saliva of *Ligus lineolaris*, a hemipteran insect pest, which interacts in extra-oral digestion of plant material prior to the uptake, was found to quickly digest dsRNA (Allen and Walker, 2012). Supporting this idea, Christiaens et al. (2014) recently proved that the dsRNA is degraded by dsRNases in the salivary secretions and also in the hemolymph of the pea aphid, *Acyrthosiphon pisum*. The presence of dsRNases in the midgut makes the insect recalcitrant to RNAi by oral feeding. The pest desert locust, *Schistocerca gregaria*, expresses dsRNases in the midgut (Wynant et al., 2014b). *S. gregaria* is recalcitrant to ingested dsRNA, whereas, Wynant et al. (2012) showed an effective systemic RNAi-response to injected dsRNA. Supporting this hypothesis, Garbutt (2011) and Garbutt et al. (2013) noted that dsRNAs are rapidly degraded in the hemolymph of RNAi recalcitrant lepidopteran *Manduca sexta*, but not in the RNAi sensitive cockroach *Blatella germanica*.

The presence of viruses in the hemolymph of insects may also act as an important barrier for RNAi efficiency. The siRNA pathway is in essence an anti-viral mechanism in many plants and animals. These viruses can interfere with the efficiency of siRNAs by saturating the RNAi core machinery, as demonstrated for vertebrate studies (Kanasty et al., 2012). The co-evolution between these viruses and RNAi defense has also led to the development of RNAi-blocking proteins called viral suppressors of RNA silencing (VSRs) in some viruses (Haasnoot et al., 2007). Lepidopterans are abundant in specific viruses in the hemolymph (Garbutt, 2011), which may be an additional factor that explains why most are recalcitrant to RNAi, besides the harsh conditions in the gut for dsRNA. Similarly, for insects, a hypothetical idea has shown that viruses can interfere with the RNAi mechanism in many ways, for example, by producing RNAi suppressor genes and/or RNA decoys, and manipulation of host gene expression. This has been reviewed in the past by Swevers et al. (2013).

### Length and concentration of dsRNA

Several important questions arise when designing RNAi experiments, including the length and concentration of dsRNA. Here, it is important to remember that dsRNA can induces RNAi through siRNAs, and afterwards yielding a wide range of siRNAs which might influence the potential for off target effects. The processing of dsRNA does not occur at fixed/phased ~21 nt intervals (Nandety et al., 2015). The dsRNA sequence recognized as foreign can be cleaved by Dicer beginning at basically any nucleotide, generating a number of potentially overlapping siRNA ranging in size and sequence that are further populated by other size siRNAs generated by the RNAi mechanism. Indeed, the diversity of formed siRNA is enormous and with potential for great homology to siRNA/dsRNA of different target mRNAs, therefore increasing the chances for off-target and non-targeted effects.

The length and optimal concentration of exogenous dsRNA are very important for effective RNAi. The required length of dsRNA to attain an effective RNAi will vary relying on insect species (Bolognesi et al., 2012). A study demonstrated that 60 and 30-bp dsRNAs induce 70 and 30% of gene knockdown in *Tribolium*, respectively (Miller et al., 2012). However, most of the studies reported that dsRNA ranged from 140 to 500 nucleotides in length are required for successful RNAi and some reported success using a dsRNA of 1842 nucleotides (reviewed in Huvenne and Smagghe, 2010). As recently reviewed (Andrade and Hunter, 2016), dsRNA longer than 200 nucleotides after dicer cleavage results in many siRNAs, which contributes to the RNAi response as well as prevents the resistance due to the polymorphism variation encoded by nucleotide sequence. Additionally, so far it is not clear on what region of the gene (coding region, 3′ or 5′ end) is ideal for dsRNA design. In the pea aphid *A. pisum*, no difference in mortality was observed in groups of insect fed with dsRNA matching the 5′ or 3′ end of the hunchback (hb) gene (Mao and Zeng, 2012 and reviewed by Andrade and Hunter, 2016). An apoptosis gene from the mosquito *Aedes aegypti, AaeIAP1*, was knocked down more efficiently when dsRNA targeting the 3′ end was used (Pridgeon et al., 2008). Nevertheless, there is a consensus among insect researchers that there is need for a screening of several dsRNAs of a certain gene, and that the dsRNA can be designed to be highly specific to the target gene and insect species, or designed to have a broader spectrum toward several closely related species (Whyard et al., 2009; Noh et al., 2012; Andrade and Hunter, 2016).

The optimal concentration has to be determined for every target gene and organism in order to induce silencing. It is not true that surpassing that optimal concentration necessarily leads to higher silencing (Meyering-Vos and Müller, 2007; Shakesby et al., 2009). In addition, when multiple dsRNAs are injected, competition will happen in cellular uptake between dsRNAs and also oversaturation can occur, resulting in a poor RNAi response (Parrish et al., 2000; Barik, 2006; Miller et al., 2012). Baum et al. (2007) reported that dsRNA targeting *V-ATPase* from CPB also caused silencing in the western corn rootworm in a concentration-dependent manner. Oversaturation of the components that are involved in the siRNA and miRNA pathways can interfere with the miRNA pathway leading to phenotypes related with the loss of the miRNA function. This restraint might lead to lethality, since miRNAs are important for growth and development (Grimm et al., 2006; Tomoyasu et al., 2008).

## Toward insect pest control: improving RNAi efficiency

### Nanoparticles

The efficiency of RNAi is mainly driven by the delivery/uptake of intact dsRNA into cells. Therefore, nanoparticles can be used to reduce dsRNA degradation and to increase the cellular uptake of intact dsRNA. Polymeric nanoparticles are produced using natural and synthetic polymers by wet synthetic routes. These are used because of their stability, ease for surface modification (Vauthier et al., 2003; Herrero-Vanrell et al., 2005) as well as their biodegradability and environmental safety. Zhang et al. (2010) used the polymer chitosan to encapsulate dsRNA and achieve RNAi in mosquitoes. Chitosan nanoparticles are designed by self-accumulation of polycations with dsRNA via electrostatic forces among positive and negative charges of the amino groups in the chitosan and phosphate groups on the backbone of the nucleic acid, respectively. This method is suitable with long dsRNA and siRNA. Chitosan nanoparticles were then mixed with diet and conveyed by oral ingestion to larvae. This system is somewhat low-cost, needs equipment and labor (Zhang et al., 2010), but provides high-throughput evaluation of phenotypes, including evaluation of behaviors (Mysore et al., 2013, 2014). Additionally, chitosan polymers are nontoxic and easily biodegradable (Dass and Choong, 2008). Zhang X. et al. (2015) reported that the target genes in *A. gambiae* (*AgCHS1* and *AgCHS2)* and *A. aegypti* (*sema1a*) were effectively knocked down during larval development by using chitosan nanoparticles. Additionally, He et al. (2013) reported that when newly hatched larvae of the RNAi recalcitrant lepidopteran pest, Asian corn borer (*Ostrinia furnacalis*), fed on four different treatments (diet containing the mixture of fluorescent nanoparticle (FNP) and CHT10-dsRNA; naked CHT10-dsRNA; FNP and GFP-dsRNA; and GFP-dsRNA treatments), only the larvae fed on the diet containing the mixture of FNP and CHT10-dsRNA showed clear RNAi gene silencing. These included a reduced larval size, failure to moult and eventually death. Therefore, the results clearly indicated that addition of FNP was vital in eliciting a strong enough gene knockdown.

### Liposomes

The therapeutic application of exogenous RNA is mainly depending on the delivery vehicle that delivers the exogenous RNA safely and effectively to target cell. We believe that liposome vesicles meet these requirements (Smyth Templeton, 2002). Liposomes are composed of natural lipids and they are non-toxic and easily biodegradable (Van Rooijen and van Nieuwmegen, 1980). They are already used in drug formulations, where the drugs are enclosed in the liposome and these liposomes are then transferred without quick degradation and minimum side effects to the receivers (Gregoriadis, 1977). Liposomes are more appropriate for assessing their targetable proteins when distinguished with other drug delivers (Grislain et al., 1983; Illum et al., 1983). For example, Drosophila S2 cells lack the *Sid-1* homologous genes, but uptake of the dsRNA happens through receptor-mediated endocytosis (Saleh et al., 2006; Ulvila et al., 2006). This is a slow process, and transfection reagents are required to enhance the dsRNA delivery to gut cells. Whyard et al. (2009) demonstrated that four different species of *Drosophila* (*D. melanogaster, D. sechellia, D. yakuba*, and *D. pseudoobscura*) were selectively killed when larvae were fed on γTub23C-dsRNA encapsulated in cationic liposomes, which target the 3′ UTR of the γ-tubulin gene. None of the drosophilid species exhibited any RNAi-silencing when fed on non-encapsulated dsRNA. More recently, Taning et al. (2016) also successfully used liposomes to improve RNAi silencing in the spotted wing Drosophila (*Drosophila suzukii*). They observed that feeding naked dsRNA did not cause any mRNA silencing, while they were able to reach 40–50% silencing using Lipofectamine (Thermo Scientific). These experiments indicated that the use of liposomes was very critical to achieve RNAi in this important pest insect. Therefore, delivering dsRNA through liposomes could be an appropriate strategy in certain cases.

### Chemical modifications

Developing chemical modifications of one or both strands may be of use to improve stability, expand the half-life of the siRNA duplexes in circulation *in vivo*, increase the bio-distribution and pharmacokinetic properties of siRNAs, target siRNA to specific cells, increase the target binding affinity, and to improve drug delivery (Kurreck, 2003; Manoharan, 2003; Dorsett and Tuschl, 2004). Nevertheless, the safety concerns and cost-effectiveness have to be considered in order to use chemically modified nucleotide. One interesting application of such chemical modifications could be to increase the specificity of dsRNA. Jackson et al. (2003) reported that adding methyl-groups to the 2′ position of the ribosyl ring of the 2nd base of the siRNA could reduce such off-target effects. These siRNAs had 3′ hydroxyl groups and 5′ monophosphates and no sequence bias was detected for both 3′ and 5′ nucleotides at the cleavage site. The gene silencing was more effective when short duplexes with 3′ overhangs at each end were utilized than when the duplex was blunt ended (Elbashir et al., 2001). Most scientists still build siRNA duplexes with 3′-TT overhangs (the “Tuschl Design”) on both strands. Other designs are also feasible: for instance, siRNAs without 3′ overhangs had been active in silencing in mammalian cells (Czauderna et al., 2003), and single 3′-overhang structures in the guide strand were also active (Lorenz et al., 2004).

## Novel delivery methods

Delivery of dsRNA is a major challenge in RNAi-based plant protection method. After identifying the target gene, choosing a convenient strategy to deliver the dsRNA into the insect body is very important. Microinjection is a good strategy for functional genomic studies but this method is not suitable to control insect pests in the field. In addition, microinjection has some limitations; it is highly technical and is difficult to achieve in some species, such as small and/or aquatic species (Nunes and Simões, 2009; Walshe et al., 2009). Sprayable RNAi-based products are in the process of development and are expected to be on the market in 2017/18. These products can be divided into the following categories: (1) direct control agents, (2) resistance repressors, (3) developmental disruptors, and (4) growth enhancers (EPA, 2014). Spraying the dsRNA might be useful to control some pest population in the field, but not all. For example, Li et al. (2015a) reported that the spraying method would not affect the piercing-sucking pests feeding on phloem sap, or stem borer pests feeding in the plant stems. The dsRNA-expressing transgenic plants reduce the crop damage, but effective killing of the pest population has not yet been accomplished (Baum et al., 2007; Mao et al., 2007; Pitino et al., 2011; Kumar et al., 2012; Zhu et al., 2012).

### Root absorption and trunk injection

Delivery of dsRNA to phytophagous insects could be achieved by supplying dsRNA through root absorption or injection into plant vessels, where these insects can naturally acquire dsRNA through sucking or chewing (Andrade and Hunter, 2016; Figure 1 Left and Middle panel).

A proof of concept for *in planta* dsRNA delivery, without a transformation event was first described by Hunter et al. (2012) (recently reviewed in Andrade and Hunter, 2016). Full-sized citrus and grapevines trees were exposed to the dsRNA either by foliar spray, root drenching and trunk injections. The dsRNA could be detected in 6-years old Citrus plants (2.5 m tall) for 7 weeks post a single exposure using 2 g of dsRNA in 15 L of water. These experiments demonstrated that two hemipteran insects as well as a xylem-feeding leafhopper were also taking up the dsRNA after feeding on host plants previously treated with dsRNA. This effort clearly shows that RNAi can be archived by dsRNA supplied exogenously. The dsRNA moved through the vascular system of the citrus plant and the dsRNA can be taken up, for instance by psyllids which feed on the phloem. One example is the Asian citrus psyllid, *Diaphorina citri*, which exhibited increased mortality when fed on dsRNA for Arginine Kinase (dsRNA-AK). Moreover, the dsRNA was identified in psyllids and leafhoppers for 5/8 days post ingestion from plants, while in treated citrus dsRNA was found up to 57 days post treatment (Hunter et al., 2012). These results support the possibility to apply RNAi approaches for area-wide pest control, especially for irrigated systems and substrate grown plants such as tomato, eggplant, cucumber among other vegetables and lettuce.

**Figure 2:**
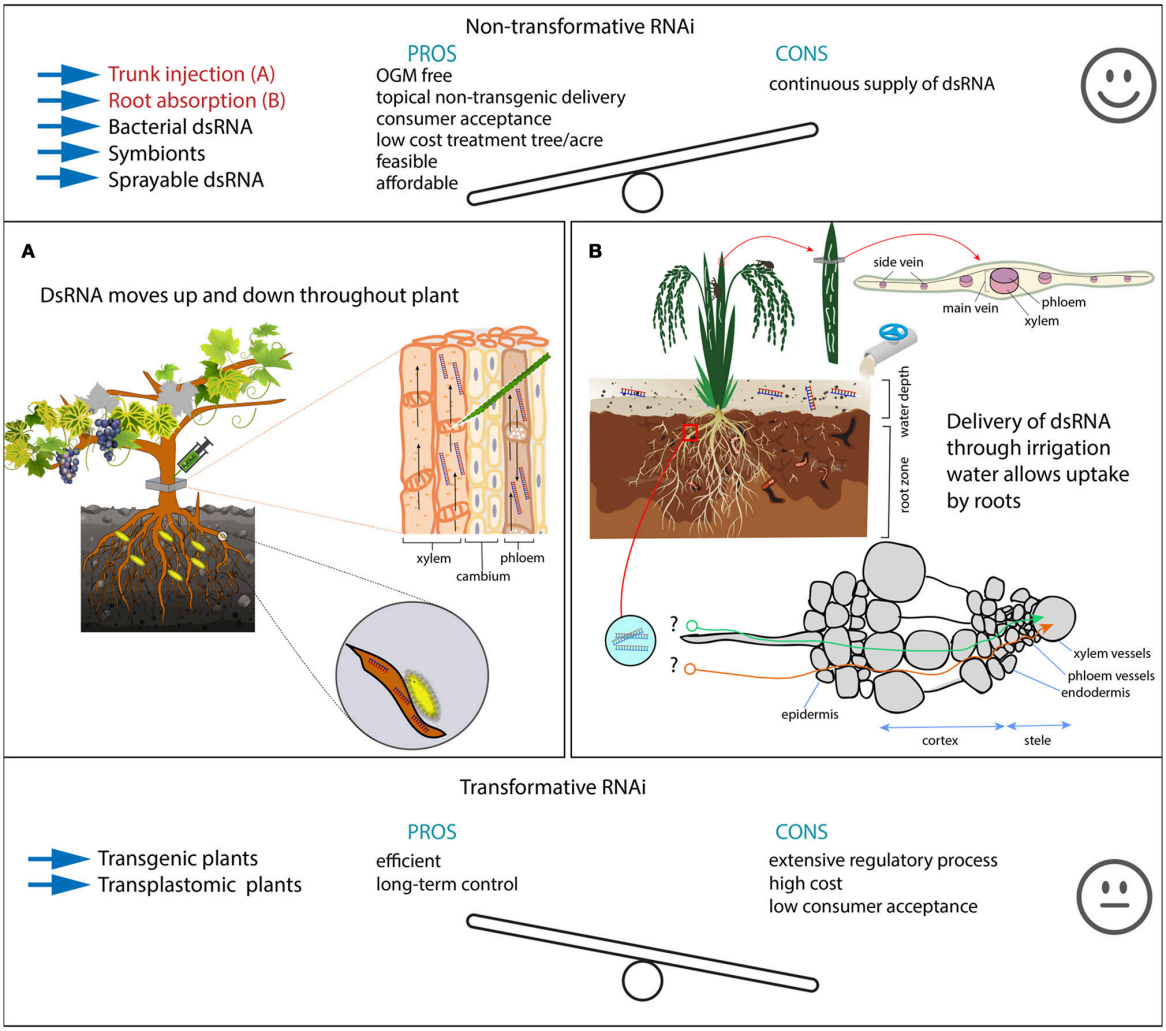
**Pros and cons of RNAi-mediated plant protection methods**. Top and bottom panel show pros and cons of non-transformative vs. transformative RNAi-mediated pest control. Methods highlighted in red are further detailed in the illustrations **(A,B)**. The middle panel shows two possible strategies for non-transformative RNAi (Hunter et al., 2012; Andrade and Hunter, 2016). Trunk injections deliver dsRNA in both vessel systems (xylem and phloem), allowing dsRNA to move up and down **(A)**. This can be particularly important for root feeding insects in perennials, where the control by using regular insecticides prove difficult and is of low efficiency. In these examples, we hypothesize the control of *Eurhizococcus brasiliensi* (Margarodidae), a hemipteran pest from vine grapes, but also it has the potential to control other insects, including aphids and chewing insects that feed on leaves and shoots. Irrigated rice is cultivated in heavy clay soils with a regular water table of ~10–15 cm **(B)**. Here, we also hypothesize that the delivery of large amounts of dsRNA though irrigation water may control rice water weevil larvae that feed on roots, but also adults that feed on leaves (see longitudinal scars indicating adult feeding). A similar experiment was carried out by Hunter et al. (2012) in citrus. However, it is unclear how dsRNA penetrates the root cells to become available into the plant vascular system **(B)**.

However, it is important to bear in mind that this strategy will demand mass-production of dsRNA, which may be costly using molecular biology kits and therefore bacterial production of dsRNA is recommended. Additionally, this system can be adapted to use new foliar shoots in 1.5 mL tubes for screening of a large number of dsRNA molecules. The newly emerged seedlings or foliar shoots absorb and deliver dsRNA through vascular tissues allowing screening of potential targets for RNAi. The shoots can remain viable for long periods (~40 days, Andrade and Hunter, 2016) making it also possible to investigate the insect biology parameters, such as eggs viability, mating, larval, and nymphal development. A similar approach could be exploited toward rice water weevils (RWW) such as *Lissorhoptrus oryzophilus* and *Oryzophagus oryzae* where larvae feed on roots and cause high yield loss by roots cutting during vegetative stages. The adults feed by scraping rice leaves. We hypothesize that the delivery of dsRNA through irrigation may penetrate through roots and control larvae and adults as well (Figure 2). This hypothesis is based on the effectiveness of this approach used by Andrade and Hunter (2016) working with coleopterans, the western corn rootworm *Diabrotica virgifera* and the root weevil *Diaprepes abbreviatus*. Adults appear in the rice field after the hibernation period, they mate in the rice field on the rice plants before females swim just below the water surface to lay eggs. Feeding scars on the leaves indicate the presence of insects, therefore, dsRNA sprayed on the leaves may kill adults before mating and before females have chance to lay eggs.

Furthermore, rice plant roots were immersed in a solution containing dsRNA targeting carboxylesterase (*Ces*) and a cytochrome P450 (*Cyp18A1*) from BPH; the target genes were knocked down and high mortality was observed when the BPH nymphs fed on treated plants. Likewise, when maize seedlings were irrigated with dsRNA of Kunitz-type trypsin inhibitors (dsKTI), this resulted in a high mortality rate with the Asian corn borer, *Ostrinia furnacalis* (Li et al., 2015a). Although interesting, Dubelman et al. (2014) reported that the soil persistency of dsRNA can be short, with a rapid breakdown within 2–3 days. Therefore, the dsRNA stability in the soil is still a matter of question.

The phloem is considered as the channel for movement of the silencing signal, carrying proteins, hormones and nucleic acids. In contrast, xylem transports water and ions and is free of RNA (Buhtz et al., 2008). The phloem sap is an RNase-free environment so the dsRNAs or siRNAs could be stable for long periods or associate with other proteins (Doering-Saad et al., 2002). However, once the silencing signal reaches the target tissue, the signal again can spread through the movement between adjacent cells. Molecular transport can happen either symplastically through plasmodesmata (i.e., the channels which connects adjacent cells) or apoplastically through a process across the cell membrane, the cell walls and intracellular movement (Melnyk et al., 2011). The size limit for molecules is about 27 kilodaltons (kDa) passing via the plasmodesmata, but plasmodesmata can alter their size and selectivity to permit entry of bigger molecules (Imlau et al., 1999). Virus-encoded proteins can change the exclusion limit of plasmodesmata and possibly there are cellular proteins acting in a similar fashion to enable the movement of bigger molecules (Melnyk et al., 2011). In conclusion, the movement of RNA from any single location can happen either within the phloem or between the cells.

From a pest control perspective, root drench/absorption may not be viable for perennial plants and old established orchards. So for these, injections in the plant trunk can deliver dsRNAs into the vascular plant systems of xylem and phloem. Trunk injection systems such as Arborjet® are available and may be used to deliver dsRNA into several plants plant species. In old established orchards such as citrus, apple, pear, peach, coffee, plum, grapevine, the control of pest insect may prove difficult, especially for root-feeding insects that live underground feeding on the roots, making the control by traditional insecticide spray impracticable. Also root drenching as demonstrated above is not viable due to the impossibility to dig up and replant again; this obviously would cause a dramatic stress in the trees, requiring several years for a complete recovery. To illustrate this, the Margarodidae is a family of scale insects that feed attached to the roots of grapevines, occurring in many countries of South America and South Africa. The decline in plant vigor becomes severe over time, leading to plant death; ultimately the growers abandon grape cultivation and move to new areas (Botton et al., 2004). A trunk injection of dsRNA targeting lethal genes from this species would provide an efficient strategy against a pest insect so far without an efficient control method. If we consider trunk injections, the dsRNA could easily reach both phloem and xylem and rapidly spreads toward the root and shoot systems (Figure 2). This strategy may prove to be more important for sap-sucking insects than to chewing insect as well as caterpillars for which feeding relies largely on leaves. Additionally, in perennial plants such as in fruit orchards, trunk-injections of dsRNA can be considered as an environment-friendly alternative to traditional sprays targeting the pest insect with no effect on natural enemies and pollinators.

### Bacteria and viruses

The delivery of dsRNA either by spraying or roots soaking results in transient (necessitating continuous or repeat exposures) presence of dsRNA in the plant tissue (Murphy et al., 2016). The delivery of dsRNA using bacteria has many advantages when compared with plant-mediated dsRNA delivery or *in vitro* synthesized dsRNA delivery. The application of bacteria-expressed dsRNA is less in cost when compared with *in vitro* synthesized dsRNA. Moreover, large-scale production of bacteria, which express dsRNA for use as pesticide, could turn into a reality soon. Persistent and large-scale delivery of dsRNA is required to kill an insect pest and also to reduce the resistance development (Huvenne and Smagghe, 2010). The bacteria-expressed dsRNA pesticides can be sprayed on crops at any time because of the ease of producing large amounts of bacteria-expressing dsRNAs. Initially, the recombinant *Escherichia coli* was engineered for dsRNAs production and fed to *C. elegans* (Kamath et al., 2003). In insects, the same strategy was applied to CPB, which were fed on different *E. coli* transformations targeting five different mRNAs (Zhu et al., 2011). An RNAase II-deficient *E. coli* was used for dsRNA production; after beetles have ingested the bacteria, significant mortality and loss of body weight were observed. More recently, a biotechnology company developed a technology called Apse RNA Containers™ (ARCs) that allows the mass-production of encapsulated dsRNA using bacteria. Plasmids coding for naturally occurring proteins such as capsids, are co-transformed with another plasmid coding for dsRNA sequences plus a “packing site.” While bacteria are growing in culture, they produce protein subunits that self-assemble around RNA in the cell, including the packing site sequence. After bacteria purification, the resulting RNA is environmentally stable and a ready-to-spray product.

Recently, the use of symbiontic bacteria has been shown to be a promising delivery strategy as well. Symbiont-mediated RNAi is an intriguing strategy in which the relationship between culturable symbiotic gut bacteria and the hosts can be exploited in order to constitutively produce dsRNA to induce RNAi in the host. The symbiont-mediated RNAi is a versatile technology to study the gene function and also a biopesticide to control the pest population. Hence, Whitten et al. (2016) reported that ingested recombinant bacteria successfully competed with the wild-type microflora in the long-lived hematophagous insect *Rhodnius prolixus* and the short-lived polyphagous insect *Frankliniella occidentalis*; also horizontally transmissible phenotypes were knocked down. In this work, the authors engineered dsRNA expression cassettes suitable for actinobacterium and proteobacterium from *R. prolixus* and *F. occidentalis*, respectively. The transformation (plasmid-producing dsRNA) of the RNaseIII-deficient bacteria allowed stable synthesis of specific dsRNA molecules, penetration in the insect gut cells and initiation of RNAi.

Yeasts are naturally growing on the surface of rotting fruits and produce volatiles, which attract Drosophilids (Becher et al., 2012; Scheidler et al., 2015). Murphy et al. (2016) reported that *D. suzukii* larval survival rate, number of eggs laid by females and locomotor activity of flies were decreased when the *D. suzukii* was fed on genetically modified *Saccharomyces cerevisiae* expressing dsRNA targeting *D. suzukii y-tubulin 23C* (*yTub23C*).

Finally, also viruses can be used as vectors for dsRNA production. Recently, plant viruses have been investigated as tools to trigger RNAi in plants (Khan et al., 2013; Wuriyanghan and Falk, 2013; Nandety et al., 2015). Generally, plants respond to infections caused by viruses through the siRNAi pathway (vsRNAs). Therefore, if an insect-specific RNAi inducer sequence is introduced into an engineered plant virus, this will produce siRNAs in the plant that are specific for insect targets (Nandety et al., 2015). The RNAi effect can be induced when insects feed on plants containing engineered virus to produced specific siRNAs.

All plant-infecting viruses move inside the plant systematically through the phloem. For that reason, the recombinant plant viruses might target the phloem-feeding insect pests. To examine this, Wuriyanghan and Falk (2013) used the recombinant *Tobacco mosaic virus* (TMV) to target the potato psyllid, *Bactericera cockerelli*. The mRNA abundance and also the progeny production in the psyllids were decreased when fed on tomatillo plants infected with recombinant TMV harboring *B. cockerelli actin* and *V-ATPase* sequences. Likewise, the fecundity of citrus mealybug, *Planococcus citri*, was reduced and mortality increased when fed on *Nicotiana benthamiana* plants infected with recombinant TMV (Khan et al., 2013).

Controlling insects through recombinant plant virus-produced dsRNA is particularly interesting for use in woody plants such as citrus, grapes, coffee, apple due to the difficulty and time to produce transgenic plants. Also for old established orchards, vineyards that need to be protected from several insect pests, including sap- and root-feeders, this technique could be of interest. Transgenic plants expressing dsRNA would prove very difficult in these cases. Therefore, recombinant viruses to deliver dsRNA might be possible and attractive with similar results and efficiency of conventional transformative methods (Figure 2). Similarly, insects are known to harbor a variety of viruses that are species-specific including baculoviruses, picornaviruses, and parvoviruses among many others. Then these viruses could be engineered to express specific dsRNAs and delivered to insect populations directly (Swevers et al., 2013).

### Transplastomic plants

In insects, it is largely accepted that the RNAi machinery is triggered by the presence of dsRNA. Long dsRNAs are required for efficient uptake and biological activity in the insect pest (Bolognesi et al., 2012). However, the dsRNAs expressed *in planta* is diced into siRNAs (Kumar et al., 2009), which afterward is ingested by insects. This might lead to a limited RNAi-effect in many insects. To overcome this, Zhang J. et al. (2015) engineered potato plants to express dsRNA in organelles lacking RNAi processing machinery, such as chloroplasts. These chloroplasts (plastids) are derived from free-living cyanobacteria, which have no RNAi pathway, leading to an accumulation of dsRNA in these organelles. When Zhang J. et al. (2015) fed potato plants, producing ACT-dsRNA in their chloroplasts, to CPB larvae 100% larval mortality was observed, whereas no larval mortality was observed when larvae were fed on ACT-dsRNA expressing nuclear transgenic plants.

## Conclusions and future perspectives

RNAi technology has been an effective tool in functional genomics studies and its application toward pest management is already close to a reality (Kupferschmidt, 2013; Palli, 2014; Saurabh et al., 2014; Nandety et al., 2015; Sherman et al., 2015; Zotti and Smagghe, 2015; Andrade and Hunter, 2016). The effectiveness of the RNAi mechanism is mainly depending on the delivery, stability, and uptake of dsRNA by target species. Apparently, for some insects *Sid-1* like genes are involved in dsRNA uptake (Xu et al., 2013; Cappelle et al., 2016), however this gene is not ubiquitously present across insect taxa as reported by several authors (Table 1). The systemic RNAi is still a matter of investigation in insects, and so far there is no consensus on what mechanism is involved behind spreading the silencing signal. The holistic understanding of systemic properties of dsRNA along with improvements toward delivery methods is underway, and in the coming years will provide innovative breakthrough applications for management of pest insects with a unique mode of action. Furthermore, because of the high specificity as a consequence of its sequence-dependent mode of action—typically targeting a single gene—RNAi will be safer than any pesticide currently available in the marked. The high specificity reduces the negative impact produced by broad-spectrum insecticides, and preserves the natural enemies and beneficial fauna in the crop area. The beneficial fauna helps for a more efficient pollination process as well as the natural enemies help to keep the pest insect populations below economic thresholds.

Delivery of dsRNA using chemically modified molecules, polymer nanoparticles, liposomes, viruses or bacteria, could increase efficacy in attaining a potent RNAi response. The choice of the delivery method and the choice of formulation would of course depend on the circumstances, on the target insect and on the reason for impaired RNAi-efficiency. For example, liposomes and polymers could be used where a limited cellular uptake is causing the insect to be refractory to RNAi. When stability of dsRNA in the insect body is the main issue, polymer- or liposome nanoparticles and bacteria could be used. Insect virus-mediated delivery could be a solution for cellular uptake, degradation and in cases where the insect is difficult to reach, since the dsRNA would be immediately produced inside the insect cells infected with the viruses.

Transgenic plants, which express dsRNA can be a potent method to suppress insect pests selectively. However, due to the extensive regulatory process, non-transformative strategies can be used with similar efficiency (Hunter et al., 2012; Scott et al., 2013; Andrade and Hunter, 2016). Expression of dsRNA through transplastomic plants would be a preferable strategy to achieve improved results. Supply of dsRNA through irrigation water, root drench, or trunk injection would be a great strategy for pest insects, such as root feeders, for which no efficient control method is available at this moment. Moreover, the delivery of dsRNA through irrigation or trunk injections holds low environmental risks due to the rapid breakdown of dsRNAs within 2–3 days in the soil and plant debris (Petrick et al., 2013; Dubelman et al., 2014), as well as due to the localized application fashion.

The production of dsRNA for research purposes can be achieved by using molecular biology kits available commercially. However, the price per microgram of such *in vitro* synthesized dsRNA is too high to be commercially interesting. Since large amounts of dsRNA are needed, more cost-efficient methods for mass production are being developed, including bacterial production and synthetic nucleoside triphosphate (NTP) modifications (Palli, 2014). Bacteria-produced dsRNA is considered one of the most cost-effective methods, and biotech companies are investing in this production method to produce large quantities, affordable, and possible for small and large farms. Several agricultural companies are working toward improvements of low-cost production of ready-to-spray RNAi products, rather than to create genetically modified organisms that cost millions and take years, beyond many regulatory hurdles from governmental agencies and the public.

At present, one regulatory agency has announced authorization for the release of a GM crop containing an RNAi-based insect control event. On September 26th, 2016, the Canadian Food Inspection Agency (CFIA) has announced that they have approved the Monsanto MON87411 corn event, containing a *D. virgifera* dsSnf7 construct in combination with two Bt constructs, for commercialization and release. However, no clear frameworks have been developed for the regulation of RNAi-based pest control by most other regulatory agencies as far as we know. The Environmental Protection Agency (EPA) has released a white paper on the risk assessment of RNAi-based GM crops (EPA, 2014), but other agencies, such as the European Food Safety Authority (EFSA) are at this moment still in the process of gathering information in the form of systematic literature reviews. EPA mentions in their report that Bt-plant incorporated proteins (PIPs) risk assessment protocols could suffice for the RNAi technology in GM crops, but that conservative assumptions and additional testing could be required for the risk predictions. The main problem is that the field of RNAi is relatively new, and many questions regarding specificity, the fate of dsRNA in the environment, effects on non-target organisms, the use of bioinformatics in risk prediction, the required sequence homology of siRNA to its target region, etc. still remain unanswered at this moment. One other review that is worth citing here, is that of Lundgren and Duan (2013), who have summarized and discussed all the potential risks of this technology.

## Author contributions

MJ, MZ, GS, and OC contributed to the structure and the idea of the review. MJ, MZ, GS, and OC wrote the paper. MZ drew the figures. MJ, GS, and OC corrected the manuscript, and prepared the final version.

### Conflict of interest statement

The authors declare that the research was conducted in the absence of any commercial or financial relationships that could be construed as a potential conflict of interest.

## References

[B1] Agrawal N. Dasaradhi P. V. Mohammed A. Malhotra P. Bhatnagar R. K. Mukherjee S. K. (2003). RNA interference: biology, mechanism, and applications. Microbiol. Mol. Biol. Rev. 67, 657–685. 10.1128/MMBR.67.4.657-685.2003 14665679 PMC309050

[B2] Allen M. L. Walker W. B. (2012). Saliva of *Lygus lineolaris* digests double stranded ribonucleic acids. J. Insect Physiol. 58, 391–396. 10.1016/j.jinsphys.2011.12.014 22226823

[B3] Alves A. P. Lorenzen M. D. Beeman R. W. Foster J. E. Siegfried B. D. (2010). RNA interference as a method for target-site screening in the western corn rootworm, *Diabrotica virgifera virgifera*. J. Insect Sci. 10, 162. 10.1673/031.010.14122 21067417 PMC3395163

[B4] Andrade C. E. Hunter W. B. (2016). RNA Interference – Natural Gene-Based Technology for Highly Specific Pest Control (HiSPeC) in RNA Interference, ed Abdurakhmonov I. Y. (Croatia: InTech), 391–409.

[B5] Arimatsu Y. Furuno T. Sugimura Y. Togoh M. Ishihara R. Tokizane M. (2007a). Purification and properties of double-stranded RNA degrading nuclease, dsRNase, from the digestive juice of the silkworm, *Bombyx mori*. J. Insect Biotechnol. Sericol. 76, 57–62. 10.11416/jibs.76.1_57

[B6] Arimatsu Y. Kotani E. Sugimura Y. Furusawa T. (2007b). Molecular characterization of a cDNA encoding extracellular dsRNase and its expression in the silkworm, *Bombyx mori*. Insect Biochem. Mol. Biol. 37, 176–183. 10.1016/j.ibmb.2006.11.004 17244546

[B7] Aronstein K. Pankiw T. Saldivar E. (2006). SID-I is implicated in systemic gene silencing in the honey bee. J. Api. Res. 45, 20–24. 10.1080/00218839.2006.11101307

[B8] Barik S. (2006). RNAi in moderation. Nat. Biotechnol. 24, 796–797. 10.1038/nbt0706-796 16841065

[B9] Baum J. A. Bogaert T. Clinton W. Heck G. R. Feldmann P. Ilagan O.. (2007). Control of coleopteran insect pests through RNA interference. Nat. Biotechnol. 25, 1322–1326. 10.1038/nbt1359 17982443

[B10] Baum J. A. Roberts J. K. (2014). Progress towards RNAi-mediated insect pest management. Adv. Insect Physiol. 47, 249–295. 10.1016/B978-0-12-800197-4.00005-1

[B11] Becher P. G. Flick G. Rozpędowska E. Schmidt A. Hagman A. Lebreton S. (2012). Yeast, not fruit volatiles mediate *Drosophila melanogaster* attraction, oviposition and development. Funct. Ecol. 26, 822–828. 10.1111/j.1365-2435.2012.02006.x

[B12] Berry B. Deddouche S. Kirschner D. Imler J. L. Antoniewski C. (2009). Viral suppressors of RNA silencing hinder exogenous and endogenous small RNA pathways in Drosophila. PLoS ONE 4:e5866. 10.1371/journal.pone.0005866 19516905 PMC2689938

[B13] Bolognesi R. Ramaseshadri P. Anderson J. Bachman P. Clinton W. Flannagan R.. (2012). Characterizing the mechanism of action of double-stranded RNA activity against western corn rootworm (*Diabrotica virgifera virgifera* LeConte). PLoS ONE 7:e47534. 10.1371/journal.pone.0047534 23071820 PMC3469495

[B14] Botton M. Schuck E. Hickel E. R. Soria S. J. (2004). Pérola-da-terra, in Pragas de Solo No Brasil, eds Salvadore J. R. Ávila C. J. Silva M. T. B. (Passo Fundo: EMBRAPA Trigo), 457–476.

[B15] Buhtz A. Springer F. Chappell L. Baulcombe D. C. Kehr J. (2008). Identification and characterization of small RNAs from the phloem of *Brassica napus*. Plant J. 53, 739–749. 10.1111/j.1365-313X.2007.03368.x 18005229

[B16] Burand J. P. Hunter W. B. (2013). RNAi: future in insect management. J. Invertebr. Pathol. 112, 68–74. 10.1016/j.jip.2012.07.012 22841639

[B17] Cappelle K. de Oliveira C. F. Van Eynde B. Christiaens O. Smagghe G. (2016). The involvement of clathrin-mediated endocytosis and two Sid-1-like transmembrane proteins in double-stranded RNA uptake in the Colorado potato beetle midgut. Insect Mol. Biol. 25, 315–323. 10.1111/imb.12222 26959524

[B18] Chen S. L. Dai S. M. Lu K. H. Chang C. (2008). Female-specific doublesex dsRNA interrupts yolk protein gene expression and reproductive ability in oriental fruit fly, *Bactrocera dorsalis* (Hendel). Insect Biochem. Molec. Biol. 38, 155–165. 10.1016/j.ibmb.2007.10.003 18207077

[B19] Christensen J. Litherland K. Faller T. van de Kerkhof E. Natt F. Hunziker J.. (2013). Metabolism studies of unformulated internally [3H]- labeled short interfering RNAs in mice. Drug Metab. Dispos. 41, 1211–1219. 10.1124/dmd.112.050666 23524663

[B20] Christiaens O. Sweveres L. Smagghe G. (2014). DsRNA degradation in the pea aphid (*Acyrthosiphon pisum*) associated with lack of response in RNAi feeding and injection assay. Peptides 53, 307–314. 10.1016/j.peptides.2013.12.014 24394433

[B21] Czauderna F. Fechtner M. Dames S. Aygün H. Klippel A. Pronk G. J.. (2003). Structural variations and stabilizing modifications of synthetic siRNAs in mammalian cells. Nucleic Acids Res. 31, 2705–2716. 10.1093/nar/gkg393 12771196 PMC156727

[B22] Dass C. R. Choong P. F. (2008). Chitosan-mediated orally delivered nucleic acids: a gutful of gene therapy. J. Drug Target. 16, 257–261. 10.1080/10611860801900801 18446603

[B23] Doering-Saad C. Newbury H. J. Bale J. S. Pritchard J. (2002). Use of aphid stylectomy and RT-PCR for the detection of transporter mRNAs in sieve elements. J. Exp. Bot. 53, 631–637. 10.1093/jexbot/53.369.631 11886882

[B24] Dorsett Y. Tuschl T. (2004). siRNAs: applications in functional genomics and potential as therapeutics. Nat. Rev. Drug Discov. 3, 318–329. 10.1038/nrd1345 15060527

[B25] Dubelman S. Fischer J. Zapata F. Huizinga K. Jiang C. Uffman J.. (2014). Environmental fate of double-stranded RNA in agricultural soils. PLoS ONE 9:e93155. 10.1371/journal.pone.0093155 24676387 PMC3968063

[B26] Elbashir S. M. Lendeckel W. Tuschl T. (2001). RNA interference is mediated by 21- and 22-nucleotide RNAs. Genes Dev. 15, 188–200. 10.1101/gad.862301 11157775 PMC312613

[B27] El Halim H. M. A. Alshukri B. M. H. Ahmad M. S. Nakasu E. Y. T. Awwad M. H. Salama E. M.. (2016). RNAi-mediated knockdown of the voltage gated sodium ion channel TcNav causes mortality in *Tribolium castaneum*. Sci. Rep. 6:29301. 10.1038/srep29301 27411529 PMC4944135

[B28] EPA (2014). RNAi Technology as a Pesticide: Problem Formulation for Human Health and Ecological Risk Assessment. Submitted to the FIFRA Scientific Advisory Panel.

[B29] Fire A. Albertson D. Harrison S. W. Moerman D. G. (1991). Production of antisense RNA leads to effective and specific inhibition of gene expression in *C. elegans* muscle. Development 113, 503–514. 1782862 10.1242/dev.113.2.503

[B30] Fire A. Xu S. Montgomery M. K. Kostas S. A. Driver S. E. Mello C. C. (1998). Potent and specific genetic interference by double-stranded RNA in *Caenorhabditis elegans*. Nature 391, 806–811. 10.1038/35888 9486653

[B31] Furusawa T. Takayama E. Ishihara R. Hayashi Y. (1993). Double-stranded ribonuclease activity in the digestive juice and midgut of the silkworm, *Bombyx mori*. Comp. Biochem. Physiol. Part A Mol. Integr. Physiol. 104, 795–801. 10.1016/0305-0491(93)90215-q

[B32] Garbian Y. Maori E. Kalev H. Shafir S. Sela I. (2012). Bidirectional transfer of RNAi between honey bee and Varroa destructor: Varroa gene silencing reduces Varroa population. PLoS Pathog. 8:e1003035. 10.1371/journal.ppat.1003035 23308063 PMC3534371

[B33] Garbutt J. S. (2011). RNA Interference in Insects, Persistence and Uptake of Double- Stranded RNA and Activation of RNAi Genes, Doctoral dissertation, University of Bath.

[B34] Garbutt J. S. Bellés X. Richards E. H. Reynolds S. E. (2013). Persistence of double-stranded RNA in insect hemolymph as a potential determiner of RNA interference success, Evidence from *Manduca sexta* and *Blattella germanica*. J. Insect Physiol. 59, 171–178. 10.1016/j.jinsphys.2012.05.013 22664137

[B35] Garlapow M. E. Huang W. Yarboro M. T. Peterson K. R. Mackay T. F. (2015). Quantitative genetics of food intake in *Drosophila melanogaster*. PLoS ONE 10:e0138129. 10.1371/journal.pone.0138129 26375667 PMC4574202

[B36] Gordon K. H. Waterhouse P. M. (2007). RNAi for insect-proof plants. Nat. Biotechnol. 25, 1231–1232. 10.1038/nbt1107-1231 17989682

[B37] Gregoriadis G. (1977). Targeting of drugs. Nature 265, 407–411. 10.1038/265407a0 834290

[B38] Grimm D. Streetz K. L. Jopling C. L. Storm T. A. Pandey K. Davis C. R.. (2006). Fatality in mice due to oversaturation of cellular microRNA/short hairpin RNA pathways. Nature 441, 537–541. 10.1038/nature04791 16724069

[B39] Grislain L. Couvreur P. Lenaerts V. Roland M. Deprez-Decampeneere D. Speiser P. (1983). Pharmacokinetics and distribution of a biodegradable drug-carrier. Int. J. Pharm. 15, 335–345. 10.1016/0378-5173(83)90166-7

[B40] Gu L. Knipple D. C. (2013). Recent advances in RNA interference research in insects: implications for future insect pest management strategies. Crop Prot. 45, 36–40. 10.1016/j.cropro.2012.10.004

[B41] Haasnoot J. Westerhout E. M. Berkhout B. (2007). RNA interference against viruses: strike and counterstrike. Nat. Biotechnol. 25, 1435–1443. 10.1038/nbt1369 18066040 PMC7096910

[B42] He B. Chu Y. Yin M. Müllen K. An C. Shen J. (2013). Fluorescent nanoparticle delivered dsRNA toward genetic control of insect pests. Adv. Mater. Weinheim. 25, 4580–4584. 10.1002/adma.201301201 23794475

[B43] Hegedus D. Erlandson M. Gillott C. Toprak U. (2009). New insights into peritrophic matrix synthesis, architecture, and function. Annu. Rev. Entomol. 54, 285–302. 10.1146/annurev.ento.54.110807.090559 19067633

[B44] Herrero-Vanrell R. Rincón A. C. Alonso M. Reboto V. Molina-Martinez I. T. Rodríguez-Cabello J. C. (2005). Self-assembled particles of an elastin-like polymer as vehicles for controlled drug release. J. Control. Release 102, 113–122. 10.1016/j.jconrel.2004.10.001 15653138

[B45] Hunter W. B. Glick E. Paldi N. Bextine B. R. (2012). Advances in RNA interference: dsRNA treatment in trees and grapevines for insect pest suppression. Southwest. Entomol. 37, 85–87. 10.3958/059.037.0110

[B46] Huvenne H. Smagghe G. (2010). Mechanisms of dsRNA uptake in insects and potential of RNAi for pest control: a review. J. Insect Physiol. 56, 227–235. 10.1016/j.jinsphys.2009.10.004 19837076

[B47] Illum L. Jones P. D. E. Kreuter J. Baldwin R. W. Davis S. S. (1983). Adsorption of monoclonal antibodies to polyhexylcyanoacrylate nanoparticles and subsequent immunospecific binding to tumour cells *in vitro*. Int. J. Pharm. 17, 65–76. 10.1016/0378-5173(83)90019-4

[B48] Imlau A. Truernit E. Sauer N. (1999). Cell-to-cell and long-distance trafficking of the green fluorescent protein in the phloem and symplastic unloading of the protein into sink tissues. Plant Cell 11, 309–322. 10.1105/tpc.11.3.309 10072393 PMC144181

[B49] Jackson A. L. Bartz S. R. Schelter J. Kobayashi S. V. Burchard J. Mao M.. (2003). Expression profiling reveals off-target gene regulation by RNAi. Nat. Biotechnol. 21, 635–637. 10.1038/nbt831 12754523

[B50] Jarosch A. Moritz R. F. (2011). Systemic RNA-interference in the honeybee *Apis mellifera*: tissue dependent uptake of fluorescent siRNA after intra-abdominal application observed by laser-scanning microscopy. J. Insect Physiol. 57, 851–857. 10.1016/j.jinsphys.2011.03.013 21439290

[B51] Kamath R. S. Fraser A. G. Dong Y. Poulin G. Durbin R. Gotta M.. (2003). Systematic functional analysis of the *Caenorhabditis elegans* genome using RNAi. Nature 421, 231–237. 10.1038/nature01278 12529635

[B52] Kanasty R. L. Whitehead K. A. Vegas A. J. Anderson D. G. (2012). Action and reaction: the biological response to siRNA and its delivery vehicles. Mol. Ther. 20, 513–524. 10.1038/mt.2011.294 22252451 PMC3293611

[B53] Katoch R. Thakur N. (2012). Insect gut nucleases: a challenge for RNA interference mediated insect control strategies. Int. J. Biochem. Biotechnol. 1, 198–203.

[B54] Khan A. M. Ashfaq M. Kiss Z. Khan A. A. Mansoor S. Falk B. W. (2013). Use of recombinant Tobacco mosaic virus to achieve RNA interference in plants against the Citrus Mealybug, *Planococcus citri* (Hemiptera: Pseudococcidae). PLoS ONE 8:e73657. 10.1371/journal.pone.0073657 24040013 PMC3767618

[B55] Kumar M. Gupta G. P. Rajam M. V. (2009). Silencing of acetylcholinesterase gene of *Helicoverpa armigera* by siRNA affects larval growth and its life cycle. J. Insect Physiol. 55, 273–278. 10.1016/j.jinsphys.2008.12.005 19135057

[B56] Kumar P. Pandit S. S. Baldwin I. T. (2012). *Tobacco rattle virus* vector: a rapid and transient means of silencing *Manduca sexta* genes by plant mediated RNA interference. PLoS ONE 7:e31347. 10.1371/journal.pone.0031347 22312445 PMC3270032

[B57] Kupferschmidt K. (2013). A lethal dose of RNA. Science 341, 732–733. 10.1126/science.341.6147.732 23950525

[B58] Kurreck J. (2003). Antisense technologies: improvement through novel chemical modifications. Eur. J. Biochem. 270, 1628–1644. 10.1046/j.1432-1033.2003.03555.x 12694176

[B59] Lehane M. J. (1997). Peritrophic matrix structure and function. Annu. Rev. Entomol. 42, 525–550. 10.1146/annurev.ento.42.1.525 15012322

[B60] Li H. Guan R. Guo H. Miao X. (2015a). New insights into an RNAi approach for plant defence against piercing-sucking and stem-borer insect pests. Plant Cell Environ. 38, 2277–2285. 10.1111/pce.12546 25828885

[B61] Li H. Khajuria C. Rangasamy M. Gandra P. Fitter M. Geng C. (2015b). Long dsRNA but not siRNA initiates RNAi in western corn rootworm larvae and adults. J. Appl. Entomol. 139, 432–445. 10.1111/jen.12224

[B62] Li J. Wang X. P. Wang M. Q. Ma W. H. Hua H. X. (2013). Advances in the use of the RNA interference technique in Hemiptera. Insect Sci. 20, 31–39. 10.1111/j.1744-7917.2012.01550.x 23955823

[B63] Li X. Dong X. Zou C. Zhang H. (2015c). Endocytic pathway mediates refractoriness of insect Bactrocera dorsalis to RNA interference. Sci. Rep. 5:8700. 10.1038/srep08700 25731667 PMC4346973

[B64] Liu J. Smagghe G. Swevers L. (2013). Transcriptional response of *BmToll9-1* and RNAi machinery genes to exogenous dsRNA in the midgut of *Bombyx mori*. J. Insect Physiol. 59, 646–654. 10.1016/j.jinsphys.2013.03.013 23602829

[B65] Lorenz C. Hadwiger P. John M. Vornlocher H. P. Unverzagt C. (2004). Steroid and lipid conjugates of siRNAs to enhance cellular uptake and gene silencing in liver cells. Bioorg. Med. Chem. Lett. 14, 4975–4977. 10.1016/j.bmcl.2004.07.018 15341962

[B66] Lundgren J. G. Duan J. J. (2013). RNAi-based insecticidal crops: potential effects on non-target species. Bioscience 63, 657–665. 10.1525/bio.2013.63.8.8

[B67] Luo Y. Wang X. Yu D. Chen B. Kang L. (2013). Differential responses of migratory locusts to systemic RNA interference via double-stranded RNA injection and feeding. Insect Mol. Biol. 22, 574–583. 10.1111/imb.12046 23869949

[B68] Luo Y. Wang X. Yu D. Kang L. (2012). The SID-1 double-stranded RNA transporter is not required for systemic RNAi in the migratory locust. RNA Biol. 9, 663–671. 10.4161/rna.19986 22614832

[B69] Manoharan M. (2003). RNA interference and chemically modified siRNAs. Nucleic Acids Res. Suppl. 3, 115–116. 10.1093/nass/3.1.115 14510407

[B70] Mao J. Zeng F. (2012). Feeding-based RNA interference of a gap gene is lethal to the pea aphid, *Acyrthosiphon pisum*. PLoS ONE 7:e48718. 10.1371/journal.pone.0048718 23144942 PMC3492414

[B71] Mao Y. B. Cai W. J. Wang J. W. Hong G. J. Tao X. Y. Wang L. J.. (2007). Silencing a cotton bollworm P450 monooxygenase gene by plant-mediated RNAi impairs larval tolerance of gossypol. Nat. Biotechnol. 25, 1307–1313. 10.1038/nbt1352 17982444

[B72] McEwan D. L. Weisman A. S. Hunter C. P. (2012). Uptake of extracellular double-stranded RNA by SID-2. Mol. Cell 47, 746–754. 10.1016/j.molcel.2012.07.014 22902558 PMC3488460

[B73] Melnyk C. W. Molnar A. Baulcombe D. C. (2011). Intercellular and systemic movement of RNA silencing signals. EMBO J. 30, 3553–3563. 10.1038/emboj.2011.274 21878996 PMC3181474

[B74] Meyering-Vos M. Müller A. (2007). RNA interference suggests sulfakinins as satiety effectors in the cricket *Gryllus bimaculatus*. J. Insect Physiol. 53, 840–848. 10.1016/j.jinsphys.2007.04.003 17560597

[B75] Miller S. C. Miyata K. Brown S. J. Tomoyasu Y. (2012). Dissecting systemic RNA interference in the red flour beetle *Tribolium castaneum*: parameters affecting the efficiency of RNAi. PLoS ONE 7:e47431. 10.1371/journal.pone.0047431 23133513 PMC3484993

[B76] Miyata K. Ramaseshadri P. Zhang Y. Segers G. Bolognesi R. Tomoyasu Y. (2014). Establishing an *in vivo* assay system to identify components involved in environmental RNA interference in the Western Corn Rootworm. PLoS ONE 9:e101661. 10.1371/journal.pone.0101661 25003334 PMC4086966

[B77] Murphy K. A. Tabuloc C. A. Cervantes K. R. Chiu J. C. (2016). Ingestion of genetically modified yeast symbiont reduces fitness of an insect pest via RNA interference. Sci. Rep. 6. 10.1038/srep22587 26931800 PMC4773866

[B78] Mysore K. Andrews E. Li P. Duman-Scheel M. (2014). Chitosan/siRNA nanoparticle targeting demonstrates a requirement for single-minded during larval and pupal olfactory system development of the vector mosquito *Aedes aegypti*. BMC Dev. Biol. 14:1. 10.1186/1471-213X-14-9 24552425 PMC3936921

[B79] Mysore K. Flannery E. M. Tomchaney M. Severson D. W. Duman-Scheel M. (2013). Disruption of *Aedes aegypti* olfactory system development through chitosan/siRNA nanoparticle targeting of semaphorin-1a. PLoS Negl. Trop. Dis. 7:e2215. 10.1371/journal.pntd.0002215 23696908 PMC3656119

[B80] Nandety R. S. Kuoy Y. W. Nouriy S. Falk B. W. (2015). Emerging strategies for RNA interference (RNAi) applications in insects. Bioengineered 6, 8–19. 10.4161/21655979.2014.979701 25424593 PMC4601220

[B81] Noh M. Y. Beeman R. W. Arakane Y. (2012). RNAi-based functional genomics in *Tribolium castaneum* and possible applications for controlling insect pests. Entomol. Res. 42, 1–10. 10.1111/j.1748-5967.2011.00437.x

[B82] Nuez I. Félix M. A. (2012). Evolution of susceptibility to ingested double-stranded RNAs in *Caenorhabditis nematodes*. PLoS ONE 7:e29811. 10.1371/journal.pone.0029811 22253787 PMC3256175

[B83] Nunes F. M. Simões Z. L. (2009). A non-invasive method for silencing gene transcription in honeybees maintained under natural conditions. Insect Biochem. Mol. Biol. 39, 157–160. 10.1016/j.ibmb.2008.10.011 19049870

[B84] Palli S. R. (2014). RNA interference in Colorado potato beetle: steps toward development of dsRNA as a commercial insecticide. Curr. Opin. Insect Sci. 6, 1–8. 10.1016/j.cois.2014.09.011 26705514 PMC4688004

[B85] Parrish S. Fleenor J. Xu S. Mello C. Fire A. (2000). Functional anatomy of a dsRNA trigger: differential requirement for the two trigger strands in RNA interference. Mol. Cell 6, 1077–1087. 10.1016/S1097-2765(00)00106-4 11106747

[B86] Pecot C. V. Calin G. A. Coleman R. L. Lopez-Berestein G. Sood A. K. (2011). RNA interference in the clinic: challenges and future directions. Nat. Rev. Cancer 11, 59–67. 10.1038/nrc2966 21160526 PMC3199132

[B87] Petrick J. S. Brower-Toland B. Jackson A. L. Kier L. D. (2013). Safety assessment of food and feed from biotechnology-derived crops employing RNA-mediated gene regulation to achieve desired traits: a scientific review. Regul. Toxicol. Pharmacol. 66, 167–176. 10.1016/j.yrtph.2013.03.008 23557984

[B88] Pitino M. Coleman A. D. Maffei M. E. Ridout C. J. Hogenhout S. A. (2011). Silencing of aphid genes by dsRNA feeding from plants. PLoS ONE 6:e25709. 10.1371/journal.pone.0025709 21998682 PMC3187792

[B89] Price D. R. Gatehouse J. A. (2008). RNAi-mediated crop protection against insects. Trends Biotechnol. 26, 393–400. 10.1016/j.tibtech.2008.04.004 18501983

[B90] Pridgeon J. W. Zhao L. Becnel J. J. Strickman D. A. Clark G. G. Linthicum K. J. (2008). Topically applied AaeIAP1 double-stranded RNA kills female adults of *Aedes aegypti*. J. Med. Entomol. 45, 414–420. 10.1603/0022-2585(2008)45[414:TAADRK]2.0.CO;2 18533434

[B91] Rangasamy M. Siegfried B. D. (2012). Validation of RNA interference in western corn rootworm *Diabrotica virgifera virgifera* LeConte (Coleoptera, Chrysomelidae) adults. Pest Manag. Sci. 68, 587–591. 10.1002/ps.2301 22500293

[B92] Rodríguez-Cabrera L. Trujillo-Bacallao D. Borrás-Hidalgo O. Wright D. J. Ayra-Pardo C. (2010). RNAi-mediated knockdown of a *Spodoptera frugiperda* trypsin-like serine-protease gene reduces susceptibility to a *Bacillus thuringiensis* Cry1Ca1 protoxin. Environ. Microbiol. 12, 2894–2903. 10.1111/j.1462-2920.2010.02259.x 20545748

[B93] Saleh M. C. van Rij R. P. Hekele A. Gillis A. Foley E. O'Farrell P. H.. (2006). The endocytic pathway mediates cell entry of dsRNA to induce RNAi silencing. Nat. Cell Biol. 8, 793–802. 10.1038/ncb1439 16862146 PMC2731564

[B94] Saurabh S. Vidyarthi A. S. Prasad D. (2014). RNA interference: concept to reality in crop improvement. Planta 239, 543–564. 10.1007/s00425-013-2019-5 24402564

[B95] Scheidler N. H. Liu C. Hamby K. A. Zalom F. G. Syed Z. (2015). Volatile codes: correlation of olfactory signals and reception in *Drosophila*-yeast chemical communication. Sci. Rep. 5:14059. 10.1038/srep14059 26391997 PMC4585764

[B96] Scott J. G. Michel K. Bartholomay L. C. Siegfried B. D. Hunter W. B. Smagghe G.. (2013). Towards the elements of successful insect RNAi. J. Insect Physiol. 59, 1212–1221. 10.1016/j.jinsphys.2013.08.014 24041495 PMC3870143

[B97] Shakesby A. J. Wallace I. S. Isaacs H. V. Pritchard J. Roberts D. M. Douglas A. E. (2009). A water-specific aquaporin involved in aphid osmoregulation. Insect Biochem. Mol. Biol. 39, 1–10. 10.1016/j.ibmb.2008.08.008 18983920

[B98] Sherman J. H. Munyikwa T. Chan S. Y. Petrick J. S. Witwer K. W. Choudhuri S. (2015). RNAi technologies in agricultural biotechnology: the Toxicology Forum 40th Annual Summer Meeting. Regul. Toxicol. Pharmacol. 73, 671–680. 10.1016/j.yrtph.2015.09.001 26361858

[B99] Shukla J. N. Kalsi M. Sethi A. Narva K. E. Fishilevich E. Singh S.. (2016). Reduced stability and intracellular transport of dsRNA contribute to poor RNAi response in lepidopteran insects. RNA Biol. 10.1080/15476286.2016.1191728 27245473 PMC4962799

[B100] Silva C. P. Silva J. R. Vasconcelos F. F. Petretski M. D. Damatta R. A. Ribeiro A. F.. (2004). Occurrence of midgut perimicrovillar membranes in paraneopteran insect orders with comments on their function and evolutionary significance. Arthropod Struct. Dev. 33, 139–148. 10.1016/j.asd.2003.12.002 18089029

[B101] Sim S. Hibberd M. L. (2016). *Caenorhabditis elegans* susceptibility to gut *Enterococcus faecalis* infection is associated with fat metabolism and epithelial junction integrity. BMC Microbiol. 16:6. 10.1186/s12866-016-0624-8 26769134 PMC4714453

[B102] Smyth Templeton N. (2002). Liposomal delivery of nucleic acids *in vivo*. DNA Cell Biol. 21, 857–867. 10.1089/104454902762053828 12573046

[B103] Swevers L. Vanden Broeck J. Smagghe G. (2013). The possible impact of persistent virus infection on the function of the RNAi machinery in insects: a hypothesis. Front. Physiol. 4:319. 10.3389/fphys.2013.00319 24204347 PMC3817476

[B104] Taning C. N. T. Christiaens O. Berkvens N. Casteels H. Maes M. Smagghe G. (2016). Oral RNAi to control *Drosophila suzukii*: laboratory testing against larval and adult stages. J. Pest Sci. 89, 803–814. 10.1007/s10340-016-0736-9

[B105] Terenius O. Papanicolao A. Garbutt J. S. Eleftherianos I. Huvenne H. Kanginakudru S.. (2011). RNA interference in Lepidoptera: an overview of successful and unsuccessful studies and implications for experimental design. J. Insect Physiol. 57, 231–245. 10.1016/j.jinsphys.2010.11.006 21078327

[B106] Thompson J. D. Kornbrust D. J. Foy J. W. Solano E. C. Schneider D. J. Feinstein E.. (2012). Toxicological and pharmacokinetic properties of chemically modified siRNAs targeting p53 RNA following intravenous administration. Nucleic Acid. Ther. 22, 255–264. 10.1089/nat.2012.0371 22913596 PMC3426203

[B107] Tomoyasu Y. Miller S. C. Tomita S. Schoppmeier M. Grossmann D. Bucher G. (2008). Exploring systemic RNA interference in insects: a genome-wide survey for RNAi genes in *Tribolium*. Genome Biol. 9, 1. 10.1186/gb-2008-9-1-r10 18201385 PMC2395250

[B108] Ulvila J. Parikka M. Kleino A. Sormunen R. Ezekowitz R. A. Kocks C.. (2006). Double-stranded RNA is internalized by scavenger receptor-mediated endocytosis in Drosophila S2 cells. J. Biol. Chem. 281, 14370–14375. 10.1074/jbc.M513868200 16531407

[B109] Van Rooijen N. van Nieuwmegen R. (1980). Liposomes in immunology: multilamellar phosphatidylcholine liposomes as a simple, biodegradable and harmless adjuvant without any immunogenic activity of its own. Immunol. Commun. 9, 243–256. 10.3109/08820138009065997 7399568

[B110] Van Wielendaele P. Dillen S. Zels S. Badisco L. Vanden Broeck J. (2013). Regulation of feeding by Neuropeptide F in the desert locust, *Schistocerca gregaria*. Insect Biochem. Molec. Biol. 43, 102–114. 10.1016/j.ibmb.2012.10.002 23103541

[B111] Varkouhi A. K. Scholte M. Storm G. Haisma H. J. (2011). Endosomal escape pathways for delivery of biologicals. J. Control. Release 151, 220–228. 10.1016/j.jconrel.2010.11.004 21078351

[B112] Vauthier C. Dubernet C. Chauvierre C. Brigger I. Couvreur P. (2003). Drug delivery to resistant tumors: the potential of poly (alkyl cyanoacrylate) nanoparticles. J. Control. Release 93, 151–160. 10.1016/j.jconrel.2003.08.005 14636721

[B113] Walshe D. P. Lehane S. M. Lehane M. J. Haines L. R. (2009). Prolonged gene knockdown in the tsetse fly Glossina by feeding double stranded RNA. Insect Mol. Biol. 18, 11–19. 10.1111/j.1365-2583.2008.00839.x 19016913

[B114] Whangbo J. S. Hunter C. P. (2008). Environmental RNA interference. Trends Genet. 24, 297–305. 10.1016/j.tig.2008.03.007 18450316

[B115] Whitten M. M. A. Facey P. D. Del Sol R. Fernández-Martínez L. T. Evans M. C. Mitchell J. J.. (2016). Symbiont-mediated RNA interference in insects. Proc. Biol. Sci. 283:20160042. 10.1098/rspb.2016.0042 26911963 PMC4810840

[B116] Whyard S. Singh A. D. Wong S. (2009). Ingested double-stranded RNAs can act as species-specific insecticides. Insect Biochem. Mol. Biol. 39, 824–832. 10.1016/j.ibmb.2009.09.007 19815067

[B117] Winston W. M. Molodowitch C. Hunter C. P. (2002). Systemic RNAi in *C. elegans* requires the putative transmembrane protein SID-1. Science 295, 2456–2459. 10.1126/science.1068836 11834782

[B118] Winston W. M. Sutherlin M. Wright A. J. Feinberg E. H. Hunter C. P. (2007). *Caenorhabditis elegans* SID-2 is required for environmental RNA interference. Proc. Natl. Acad. Sci. U.S.A. 104, 10565–10570. 10.1073/pnas.0611282104 17563372 PMC1965553

[B119] Wuriyanghan H. Falk B. W. (2013). RNA interference towards the potato psyllid, *Bactericera cockerelli*, is induced in plants infected with recombinant tobacco mosaic virus (TMV). PLoS ONE 8:e66050. 10.1371/journal.pone.0066050 23824081 PMC3688868

[B120] Wynant N. Santos D. Van Wielendaele P. Vanden Broeck J. (2014a). Scavenger receptor-mediated endocytosis facilitates RNA interference in the desert locust, *Schistocerca gregaria*. Insect Mol. Biol. 23, 320–329. 10.1111/imb.12083 24528536

[B121] Wynant N. Santos D. Verdonck R. Spit J. Van Wielendaele P. Vanden Broeck J. (2014b). Identification, functional characterization and phylogenetic analysis of double stranded RNA degrading enzymes present in the gut of the desert locust, *Schistocerca gregaria*. Insect Biochem. Mol. Biol. 46, 1–8. 10.1016/j.ibmb.2013.12.008 24418314

[B122] Wynant N. Verlinden H. Breugelmans B. Simonet G. Vanden Broeck J. V. (2012). Tissue dependence and sensitivity of the systemic RNA interference response in the desert locust, *Schistocerca gregaria*. Insect Biochem. Mol. Biol. 42, 911–917. 10.1016/j.ibmb.2012.09.004 23022143

[B123] Xiao D. Gao X. Xu J. Liang X. Li Q. Yao J.. (2015). Clathrin-dependent endocytosis plays a predominant role in cellular uptake of double-stranded RNA in the red flour beetle. Insect Biochem. Mol. Biol. 60, 68–77. 10.1016/j.ibmb.2015.03.009 25863352

[B124] Xu H. J. Chen T. Ma X. F. Xue J. Pan P. L. Zhang X. C.. (2013). Genome-wide screening for components of small interfering RNA (siRNA) and micro-RNA (miRNA) pathways in the brown planthopper, *Nilaparvata lugens* (Hemiptera: Delphacidae). Insect Mol. Biol. 22, 635–647. 10.1111/imb.12051 23937246

[B125] Xu W. Han Z. (2008). Cloning and phylogenetic analysis of sid-1-like genes from aphids. J. Insect Sci. 8, 30. 10.1673/031.008.3001 20302524 PMC3061602

[B126] Zha W. Peng X. Chen R. Du B. Zhu L. He G. (2011). Knockdown of midgut genes by dsRNA-transgenic plant-mediated RNA interference in the hemipteran insect *Nilaparvata lugens*. PLoS ONE 6:e20504. 10.1371/journal.pone.0020504 21655219 PMC3105074

[B127] Zhang J. Khan S. A. Hasse C. Ruf S. Heckel D. G. Bock R. (2015). Full crop protection from an insect pest by expression of long double-stranded RNAs in plastids. Science 347, 991–994. 10.1126/science.1261680 25722411

[B128] Zhang X. Mysore K. Flannery E. Michel K. Severson D. W. Zhu K. Y. (2015). Chitosan/interfering RNA nanoparticle mediated gene silencing in disease vector mosquito larvae. J. Vis. Exp. 97:52523 10.3791/52523 PMC4401390 25867635

[B129] Zhang X. Y. Lu K. Zhou J. L. Zhou Q. (2013). Molecular characterization and gene functional analysis of Dicer-2 gene from *Nilaparvata lugens* (Hemiptera: Geometroidea). Insect Sci. 20, 61–68. 10.1111/j.1744-7917.2012.01539.x 23955826

[B130] Zhang X. Zhang J. Zhu K. Y. (2010). Chitosan/double-stranded RNA nanoparticle-mediated RNA interference to silence chitin synthase genes through larval feeding in the African malaria mosquito (*Anopheles gambiae*). Insect Mol. Biol. 19, 683–693. 10.1111/j.1365-2583.2010.01029.x 20629775

[B131] Zhao L. Yang M. Shen Q. Liu X. Shi Z. Wang S.. (2016). Functional characterization of three trehalase genes regulating the chitin metabolism pathway in rice brown planthopper using RNA interference. Sci. Rep. 6:27841. 10.1038/srep27841 27328657 PMC4916506

[B132] Zheng W. Liu Y. Zheng W. Xiao Y. Zhang H. (2015). Influence of the silencing sex-peptide receptor on *Bactrocera dorsalis* adults and offspring by feeding with ds-spr. J. Asia Pac. Entomol. 18, 477–481. 10.1016/j.aspen.2015.05.004

[B133] Zhu F. Xu J. Palli R. Ferguson J. Palli S. R. (2011). Ingested RNA interference for managing the populations of the Colorado potato beetle, *Leptinotarsa decemlineata*. Pest Manag. Sci. 67, 175–182. 10.1002/ps.2048 21061270

[B134] Zhu J. Q. Liu S. Ma Y. Zhang J. Q. Qi H. S. Wei Z. J.. (2012). Improvement of pest resistance in transgenic tobacco plants expressing dsRNA of an insect-associated gene EcR. PLoS ONE 7:e38572. 10.1371/journal.pone.0038572 22685585 PMC3369839

[B135] Zotti M. J. Smagghe G. (2015). RNAi technology for insect management and protection of beneficial insects from diseases: lessons, challenges and risk assessments. Neotrop. Entomol. 44, 197–213. 10.1007/s13744-015-0291-8 26013264

